# Peroral cholangioscopic diagnosis of refractory hemobilia caused by pancreatic arteriovenous malformation: a case report

**DOI:** 10.1055/a-2780-6714

**Published:** 2026-02-24

**Authors:** Min Xu, Yingying Li, Yanru Li, Yuwei Wang, Shuyi Zhang, Wen Li, Hao Zhang

**Affiliations:** 1 74769 Department of Gastroenterology, Union Medical Center, The First Affiliated Hospital of Nankai University, Tianjin, China

Pancreatic arteriovenous malformation is a rare vascular anomaly. The first reported case was described in 1968 by Halpern et al. 1 . Yang Zhuo et al. reported a case of failed treatment of biliary hemorrhage caused by a gastroduodenal artery malformation with fully covered biliary metal stents 2 . In this case, the patient was admitted due to “common bile duct stones” and experienced hemobilia after endoscopic retrograde cholangiopancreatography (ERCP). Peroral cholangioscopy revealed bleeding from a cystic lesion in the pancreatic portion of the common bile duct.

**Fig. 1:**
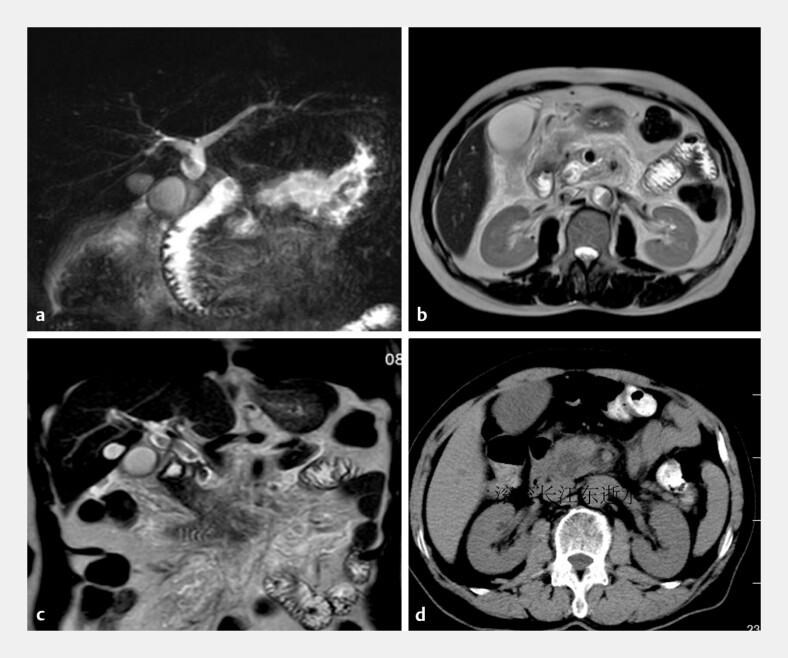
**a, b** MRCP revealing cystic dilation of the pancreatic segment of the common bile duct. **c** MRCP reveals a hypointense focus within the common bile duct. **d** Abdominal CT revealing dilation of the pancreatic segment containing hyperdensity. CT, computed tomography; MRCP, magnetic resonance cholangiopancreatography

**Fig. 2:**
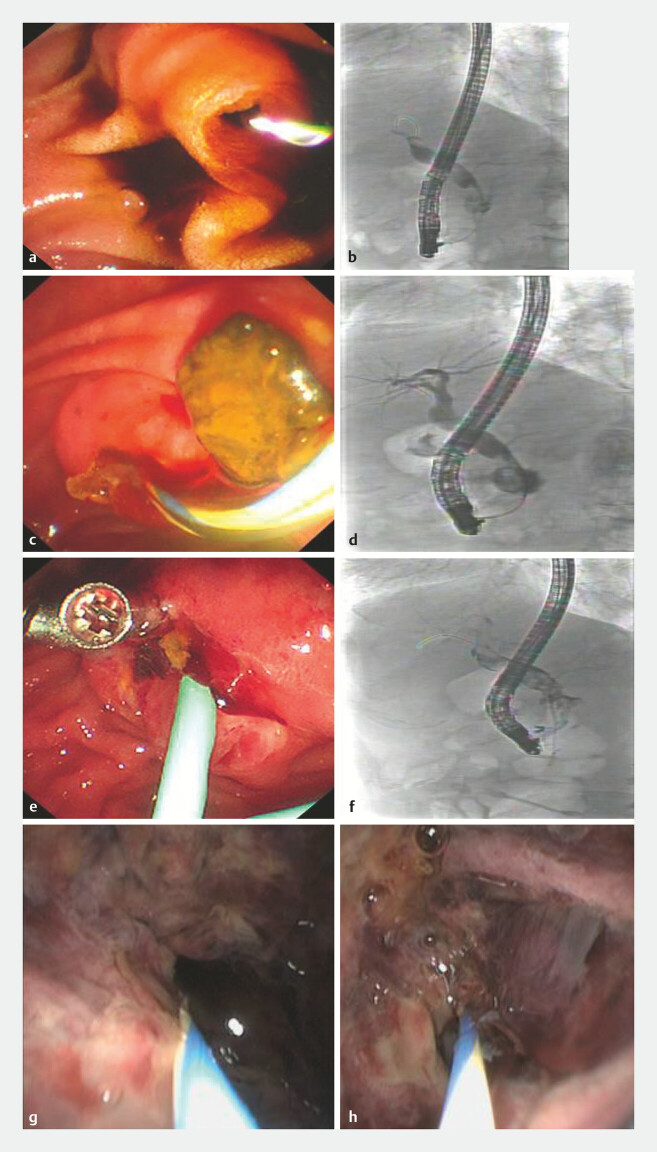
The first ERCP: **a** hemobilia from the orifice of Vater’s ampulla before cannulation. **b** ERCP reveals dilation of the extrahepatic bile duct within stones and cystic dilation of the pancreatic segment of the common bile duct. **c** Bloody bile drainage is observed after stone removal. **d** Fluoroscopy reveals a blood clot within the cystic dilation. **e** No active bleeding was observed at the EST incision margin. The second ERCP: **f** fluoroscopy reveals a blood clot within the cystic dilation. **g, h** Peroral cholangioscopy revealed a cystic dilation of the distal common bile duct. Within the cyst, the normal bile duct wall architecture was absent, and tortuous, dilated vessels along with a hematoma were observed. ERCP, endoscopic retrograde cholangiopancreatography; EST, endoscopic sphincterotomy.

A 67-year-old man was admitted to our hospital with upper abdominal pain. Magnetic resonance cholangiopancreatography showed an abnormal signal intensity in the pancreatic head, common bile duct stones and pancreatitis ( Fig. 1 ). The patient underwent ERCP to remove the stone ( Fig. 2 **a–d** ). The patient developed abdominal pain, blood in the nasobiliary drainage, and melena after ERCP. No active bleeding was observed at the endoscopic sphincterotomy incision margin ( Fig. 2 **e–f** ). Peroral cholangioscopy revealed a cystic dilation of the distal common bile duct. Within the cyst, the normal bile duct wall architecture was absent, and tortuous, dilated vessels along with a hematoma were observed ( Fig. 2 **g–h** ). The cholangioscopic findings suggested bleeding from a vascular malformation ( Video 1 ).

Peroral cholangioscopy shows a cystic dilation in the distal common bile duct with a hematoma. During DSA, the pancreatic head region exhibits dense vascularity, accompanied by rapid visualization of the portal vein. DSA, digital subtraction angiography.Video 1

**Fig. 3:**
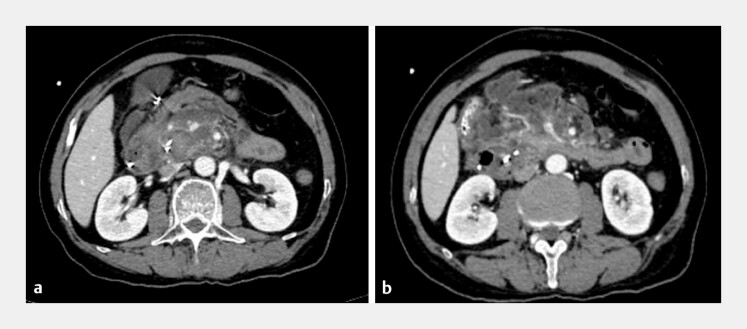
Enhanced computed tomography reveals multiple hypervascular spots in the pancreatic head and early visualization of the portal vein and superior mesenteric vein.

**Fig. 4:**
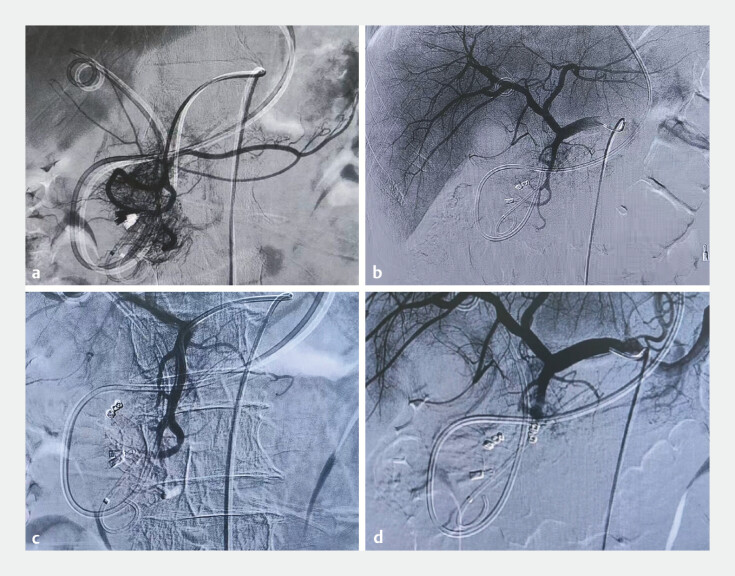
The first DSA: **a** GDA angiography: the pancreatic head region exhibits dense vascularity, accompanied by rapid visualization of the portal vein. **b** The arterioportal fistula vanished subsequent to the gelatin sponge embolization of the superior pancreaticoduodenal artery. The second DSA: **c** Angiography of the common hepatic artery still demonstrates blood supply to the pancreatic head region via the superior pancreaticoduodenal artery and **d** followed by coil embolization of the superior pancreaticoduodenal artery. DSA, digital subtraction angiography; GDA, gastroduodenal artery.

During digital subtraction angiography (DSA), angiographic findings were considered typical of an arteriovenous malformation in the pancreaticobiliary region ( Fig. 3 **a** ). After the superior pancreatoduodenal artery was embolized, post-embolization angiography confirmed the disappearance of the vascular cluster and the fistula ( Fig. 3 **b** , Video 1 ). Subsequently, the hemobilia occurred again. An abdominal contrast-enhanced computed tomographic scan showed that the surgical intervention was not feasible ( Fig. 4 **a, b** ). A repeat DSA was performed, and the superior pancreaticoduodenal artery was embolized using coils ( Fig. 4 **c, d** ). No further bleeding occurred following this procedure. The nasobiliary tube was removed 7 days later. Unfortunately, the patient died due to a cardiocerebrovascular accident.

Endoscopy_UCTN_Code_CCL_1AZ_2AN
